# Effects of Dietary *Thymus vulgaris* on Laying Hens: A Systematic Review and Meta‐Analysis of Productive Performance and Egg Quality

**DOI:** 10.1002/vms3.70614

**Published:** 2025-09-12

**Authors:** Hossein Hassanpour, Leila Nasiri, Aziz A. Fallah, Tahereh Karimi‐Shayan

**Affiliations:** ^1^ Department of Basic Sciences, Faculty of Veterinary Medicine Shahrekord University Shahrekord Iran; ^2^ Health Equity Research Center Shahed University Tehran Iran; ^3^ Department of Food Hygiene and Quality Control, Faculty of Veterinary Medicine Shahrekord University Shahrekord Iran; ^4^ Department of Biology Faculty of Science University of Mohaghegh Ardabili Ardabil Iran

**Keywords:** egg production, egg quality, laying hens, thyme

## Abstract

**Objectives:**

Natural alternatives to synthetic additives are being explored as a means to enhance laying hen performance and egg quality. The study conducted a systematic review and meta‐analysis to evaluate the effect of dietary supplementation with *Thymus vulgaris* (thyme) on productive performance and egg quality in laying hens.

**Methods:**

A comprehensive search of databases identified 18 eligible studies. Data were analysed using random‐effects models, with subgroup analyses based on the thyme form (powder, essential oil, extract) and supplementation duration (≤ 8 weeks, > 8 weeks). The unstandardized mean difference was used to calculate the effect size, and confidence intervals were generated to estimate the range of the true effect size.

**Results:**

Thyme significantly improved egg weight, egg mass, yolk colour, yolk index and shell thickness (*p* < 0.05), with very large effect sizes for egg weight and mass. However, albumen quantity decreased, while egg production rate, Haugh unit, and shell weight remained unchanged. Powdered thyme was particularly effective at enhancing egg weight and mass, while essential oils and extracts improved yolk colour. Longer supplementation periods (> 8 weeks) yielded greater benefits for egg mass and weight, whereas shorter durations (≤ 8 weeks) improved shell thickness.

**Conclusions:**

These findings suggest that thyme supplementation, particularly in powdered form and over extended periods, can enhance egg quality, offering a natural alternative to synthetic additives. This research demonstrates thyme's efficacy as a phytogenic feed additive, offering a natural solution to improve egg quality and meet the rising demand for sustainable and high‐quality poultry products.

**Summary:**

- Thyme supplementation improves egg weight, egg mass, yolk colour and shell thickness in laying hens.
- Powdered thyme shows the most significant benefits for egg weight and mass.
- Longer supplementation periods (> 8 weeks) enhance yolk colour and egg quality traits.
- Thyme offers a natural, sustainable alternative to synthetic feed additives in poultry production.

## Graphical abstract

Dietary supplementation with *Thymus vulgaris* significantly improves egg weight, egg mass, yolk colour, yolk index and shell thickness in laying hens. Powdered thyme and longer supplementation periods (> 8 weeks) yield the greatest benefits. Thyme offers a natural alternative to synthetic additives, enhancing egg quality and supporting sustainable poultry production.

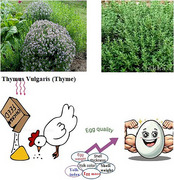

## 1 Introduction

The poultry industry plays a crucial role in meeting the growing global demand for animal protein, with eggs serving as a cost‐effective and nutritionally rich food source for human consumption (Kashyap and Goswami 2024). As the industry continues to grow, there is a pressing need to enhance the productive performance of laying hens while maintaining or improving egg quality traits, such as shell strength, yolk colour and nutritional composition (Bain et al. 2016). Traditionally, synthetic additives, including antibiotics and growth promoters, have been widely used to achieve these objectives. However, the emergence of antibiotic resistance and growing consumer awareness about food safety and natural products have prompted researchers and producers to explore alternative strategies (Al‐Harthi 2014; Prakash et al. 2024). In this context, phytogenic feed additives, derived from plants and herbs, have garnered considerable interest. This is because they offer the potential to enhance animal health, performance and product quality while avoiding the negative impacts of synthetic substances (Abdelli et al. 2021; Darmawan et al. 2022).

Among the various phytogenic additives, *Thymus vulgaris*, commonly known as thyme, has emerged as a promising candidate (Khan et al. 2012). Thyme is a perennial herb belonging to the Lamiaceae family and is widely recognized for its bioactive compounds, including thymol, carvacrol and flavonoids, which exhibit antimicrobial, antioxidant and anti‐inflammatory properties (Hammoudi Halat et al. 2022; Hassanpour et al. 2024c). These bioactive components have been demonstrated to positively impact gut health, nutrient absorption and immune function in poultry, potentially improving productive performance and egg quality (Safavipour et al. 2022) Moreover, Thyme's natural origin aligns with the growing consumer preference for clean labels and sustainable production practices, making it an attractive option for the poultry industry (Lisboa et al. 2024; Molnár and Szőllősi 2020).

Egg production is vital to the poultry industry, where multiple factors influence the overall quality and market appeal of eggs (Molnár and Szőllősi 2020). Important indicators of laying hen efficiency include the rate of egg production, egg weight and egg mass, all of which together reflect how effectively hens convert feed into eggs (Hassanpour et al. 2025). The thickness of the eggshell is a crucial characteristic, as it affects both the structural stability of the egg and helps minimize breakage during handling and transportation (Rosaiah et al. 2024). In addition to production metrics, egg quality is evaluated using several key parameters, including yolk colour, yolk index, Haugh unit, albumen quality, shell thickness and shell weight (Wijedasa et al. 2020). Yolk colour is important to consumers because it's often associated with an egg's nutrition and freshness. The yolk index, which measures the yolk's height compared to its width, helps assess its quality and freshness (Watkins 2017). The Haugh unit measures egg white freshness and overall quality. Shell thickness and weight determine the eggshell's strength, preventing breakage and keeping eggs safe during storage and transport (Abebe et al. 2023; Aboonajmi and Faridi 2020).

As consumers seek nutritious and sustainably produced eggs, adding natural supplements like thyme to poultry diets is a promising way to meet these demands. However, studies on the impact of thyme supplementation on laying hens have been inconsistent, highlighting the need for a thorough review of existing studies to clarify its effects. The duration of thyme supplementation plays a significant role in determining its effects on egg production and quality traits. Different supplementation periods in studies affect how hens absorb and use thyme's active compounds, influencing their physiological responses. The form of thyme supplementation, powder, essential oil or extract impacts its effectiveness, as each has different absorption rates and bioactive profiles, influencing productive performance and egg quality. This systematic review and meta‐analysis summarize current evidence on how thyme impacts laying hens' productive performance and egg quality. The study will investigate how thyme affects critical egg quality and production metrics, including egg production rate, egg weight, egg mass, shell thickness, yolk colour, yolk index, Haugh unit and yolk and albumen quantities. In addition, it will explore how different durations and forms of thyme supplementation influence these outcomes. This research offers insights for future studies and practical poultry industry uses, helping make egg production more sustainable and efficient.

## 2 Methods

### 2.1 Study Eligibility and Data Extraction

The research protocol was registered in the PROSPERO system with reference code CRD420251017099. Available from https://www.crd.york.ac.uk/PROSPERO/view/CRD420251017099. A comprehensive search was conducted across several major databases, including PubMed, Scopus, ISI Web of Science, ProQuest, the Scientific Information Database (SID), and MagIran, to identify all relevant published studies up to 25 March, 2025. We performed the search using the following terms: (‘thyme’ OR ‘*Thymus vulgaris*’) AND (‘hen’ OR ‘laying hens’ OR ‘layer’) AND (‘egg production’ OR ‘egg index’ OR ‘shell thickness’ OR ‘egg weight’ OR ‘yolk colour’ OR ‘yolk index’ OR ‘albumen’ OR ‘Haugh unit’ OR ‘yolk’ OR ‘shell weight’ OR ‘egg mass’ OR ‘productive performance’ OR ‘egg quality’ OR ‘egg quality traits’). To select studies for inclusion in this review, we established specific criteria. These studies employed experimental designs with laying hens, including both a control group and a group receiving thyme treatment. In addition, the studies had to assess the impact of thyme supplementation in comparison to a control, with emphasis on parameters such as egg production rate, eggshell thickness, egg weight, yolk colour grading, yolk index value, Haugh unit score and weights of albumen, yolk, shell and overall egg mass. Furthermore, the thyme used in these studies could not be combined with any other plant‐derived substances or additives. The studies also had to be published in either English or Persian. Only parameters that were reported in at least six studies were considered. Measurements were recorded at the longest duration of thyme supplementation in each study, and the data were then structured according to the format shown in Table 1.

**TABLE 1:** Specifications of experimental studies related to the genus *Thymus vulgaris* included in the meta‐analysis.

Study	Year	Forms of used Thymus	Strain of laying Hen	Dose of thymus in diet	Assessing outcome time	Sample size (bird number)	Egg outcome data
El‐Hack and Alagawany	2015	Powder	Hi‐sex Brown	3, 6, 9 g/kg	16 weeks	*C* = 24, *T*_1_ = 24, *T*_2_ = 24, *T* = 24	EM, EW, HU, YI, ST, albumin, yolk
Akbari et al.	2016	Essential oil	Lohmann LSL‐Lite	100 mg/kg	8 weeks	*C* = 30, *T* = 30	EP, EM, EW, HU, ST, YC, SW
Alagawany et al.	2017	Powder	Hi‐sex Brown	9 g/kg	16 weeks	*C* = 24, *T* = 24	EP
Bala et al.	2021	Powder	Lohmann‐Brown‐Classic	1, 5, 10 g/kg	4 weeks	*C* = 30, *T*_1_ = 30, *T*_2_ = 30, *T*_3_ = 30	EP, EM, EW, HU, ST, YC, SW
Behnamifar et al.	2015	Extract	Tetra‐SL	1 g/kg	8 weeks	*C* = 12, *T* = 12	EP, EM, EW, HU, ST, YC, SW
Bolukbasi et al.	2008	Essential oil	Lohman‐LSL hybrid	200 mg/kg	12 weeks	*C* = 16, *T* = 16	EP, EW, HU, albumin, yolk
Büyükkılıç‐Beyzi et al.	2020	Essential oil	Lohman White	300 mg/kg	10 weeks	*C* = 24, *T* = 24	EP, EW, HU, ST
Esenbuga and Ekinci	2023	Essential oil	Lohmann LS White	250 mg/kg	12 weeks	*C* = 72, *T* = 72	EP, EW, HU, YI, ST, YC, SW
Hammershøj and Steenfeldt	2012	Powder	Lohmann Silver	15 g/kg	5 weeks	*C* = 60, *T* = 60	EP, EM, EW
Keshavarz‐Motamedi et al.	2017	Extract	Lohman‐LSL	5 g/kg	8 weeks	*C* = 20, *T* = 20	EP, EM, EW
Minaei‐Javid et al.	2021	Powder	Hy‐Line w36	10, 20, 30 g/kg	14 weeks	*C* = 48, *T*_1_ = 48, *T*_2_ = 48, *T*_3_ = 48	EP, EM, EW, ST, SW, YC, HU
Mohammed et al.	2022	Powder	Hy‐line Brown	5, 10 g/kg	7 weeks	*C* = 20, *T*_1_ = 20, *T*_2_ = 20	EM, EW, HU, SW, YI, albumin, yolk
Nobakht and Mehmannavaz	2010	Powder	Hy‐Line w36	20 g/kg	12 weeks	*C* = 36, *T* = 36	EP, EM, EW, YI, YC, HU, SW, ST
Radwan Nadia et al.	2008	Powder	El‐Salaam	5, 10 g/kg	13 weeks	*C* = 30, *T*_1_ = 30, *T*_2_ = 30	EP, EM, EW, YI, YC, HU, ST, albumin, yolk
Rostami and Toghyani	2022	Powder	Native	10 g/kg	12 weeks	*C* = 30, *T* = 30	EP, EM, EW, ST, YC, HU
Soliman and Kamel	2020	Powder	White Hi‐Sex	10 g/kg	10 weeks	*C* = 15, *T* = 15	EP, EM, EW, YC, albumin, yolk
Vakili and Majidzadeh	2016	Extract	Hy‐Line W36	4 g/kg	12 weeks	*C* = 40, *T* = 40	EP, EM, EW, YI, HU, ST, SW, YC
Yalcin et al.	2020	Powder	Hy‐line Brown	10, 20 g/kg	16 weeks	*C* = 36, *T*_1_ = 36, *T*_2_ = 36	EP, EW, YI, HU, ST, albumin, yolk

Abbreviations: EM, egg mass; EP, egg production; ES, egg shell; EW, egg weight; HU, Haugh unit; ST, shell thickness; SW, shell weight; YC, yolk colour; YI, yolk index.

### 2.2 Data Analysis and Risk of Bias Assessment

Effect sizes were calculated as unstandardized mean differences with 95% confidence intervals (CIs), where narrower intervals indicate higher precision (Jané et al. 2024). Effects were interpreted via Cohen's benchmarks: small (0.2), medium (0.5), large (0.8) and very large (1.3) (Sullivan and Feinn 2012). Standard errors were converted to standard deviations for uniformity. Heterogeneity analysis employed Cochrane's *Q*‐test and *I* ^2^ statistics, categorizing variability as low (25%), moderate (50%) or high (75%) (Hassanpour et al. 2024b; Huedo‐Medina et al. 2006).

Subgroup analyses were conducted using the data from studies listed in Table 2. These analyses were categorized into two main groups: 1) the form of thyme used, which included essential oil, powder and extract; and 2) the duration of supplementation, which was divided into studies that administered thyme for 8 weeks or less and those that lasted longer than 8 weeks.

**TABLE 2:** Effect of thyme supplementation on productive performance and egg quality traits.

Outcome	No. of studies	No. of trials	Effect size (95% CI)	*p* value	*I* ^2^ (%)	*Q*‐statistics (*p*)
Egg production (%)	16	22	1.94 (−4.15, 8.03)	0.533	99.4	< 0.001
Egg weight (g)	16	26	1.41(0.55, 2.27)	< 0.001	98.8	< 0.001
Egg mass (g)	12	20	1.79 (0.79, 2.78)	< 0.001	89.9	< 0.001
Haugh unit	14	22	0.19 (−0.98, 1.35)	0.755	85.7	< 0.001
Yolk colour	10	15	0.62 (0.34, 0.89)	< 0.001	92.3	< 0.001
Yolk index	6	11	2.22 (0.61, 3.82)	0.007	99.9	< 0.001
Yolk (%)	6	11	1.24 (−0.06, 2.54)	0.061	94.7	< 0.001
Albumin (%)	6	11	−1.86 (−2.81, −0.91)	< 0.001	83.4	< 0.001
Shell thickness (mm)	12	20	0.01(0.01, 0.02)	0.003	75.4	< 0.001
Shell weight (g)	7	12	−0.07 (−0.17, 0.04)	0.229	26.5	0.184

Sensitivity analysis evaluated individual study impacts on effect sizes. Publication bias was assessed via Egger's test and adjusted using the Trim‐and‐Fill method (Hassanpour et al. 2024a). All analyses were performed in Stata 11.2 (StataCorp, College Station, TX) with statistical significance set at *p* < 0.05.

The Systematic Review Centre for Laboratory Animal Experimentation (SYRCLE) developed a specialized risk of bias (RoB) tool tailored for animal studies, which we utilized to evaluate potential biases in the studies included in this meta‐analysis. This tool comprises 10 criteria designed to detect biases across various domains, including selection, performance, detection, attrition and reporting. The ten items assessed are sequence generation, baseline characteristics, allocation concealment, random housing, blinding of caregivers and investigators, random outcome assessment, blinding of outcome assessor, incomplete outcome data, selective outcome reporting and other sources of bias. Each criterion is assessed using a scoring system where a ‘yes’ indicates a low risk of bias for that aspect, a ‘no’ signifies a high risk and an ‘unclear’ rating is assigned when the information is missing or insufficient, indicating uncertainty about the risk of bias (Hassanpour et al. 2024c; Hooijmans et al. 2014).

## 3 Results

### 3.1 Study Features

**FIGURE 1:**
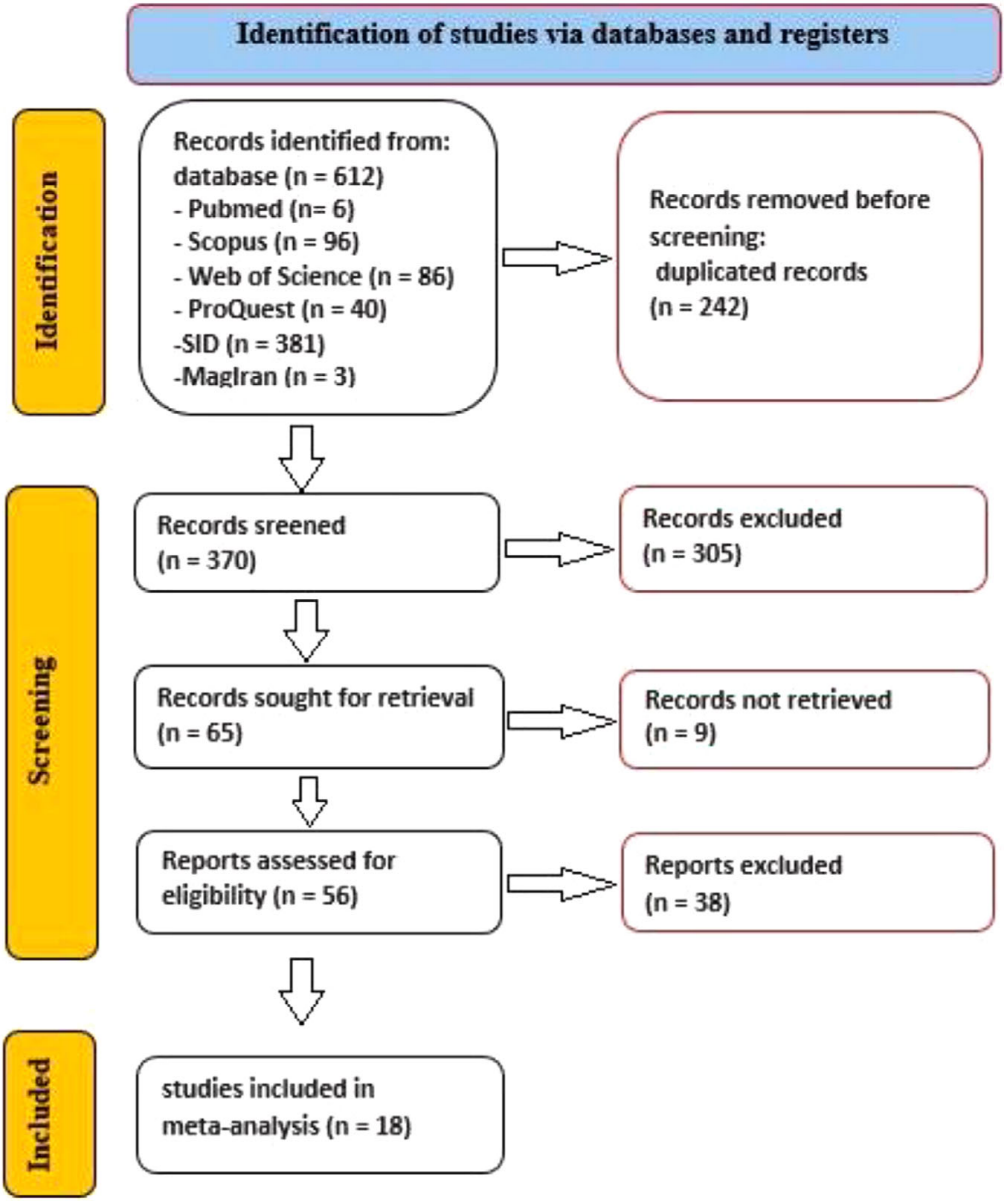
PRISMA 2020 flow diagram.

We identified 18 studies (Abd El‐Hack and Alagawany 2015; Akbari et al. 2016; Alagawany et al. 2017; Bala et al. 2021; Behnamifar et al. 2015; Bölükbaşı et al. 2008; Büyükkılıç Beyzi et al. 2020; Esenbuga and Ekinci 2023; Hammershøj and Steenfeldt 2012; Kamel 2020; Keshavarz Moetamedi et al. 2017; Minaei and Sharafi 2021; Mohammed et al. 2022; Nobakht and Mehman Navaz 2010; Radwan Nadia et al. 2008; Rostami and Toghyani 2022; Vakili and Heravi 2016; Yalçin et al. 2020) that met our criteria after removing duplicates and irrelevant entries from electronic databases (Figure 1). These studies, summarized in Table 1, primarily used powdered thyme (61.1%), followed by essential oil (22.2%) and extracts (16.7%). Thyme dosages varied: powdered thyme (1–30 g/kg), essential oil (100–300 mg/kg) and extracts (1–5 g/kg). The administration duration ranged from 4 to 16 weeks, with 33.3% of studies lasting 8 weeks or less and 66.7% extending beyond 8 weeks.

### 3.2 Evaluation of Bias Risk

**FIGURE 2:**
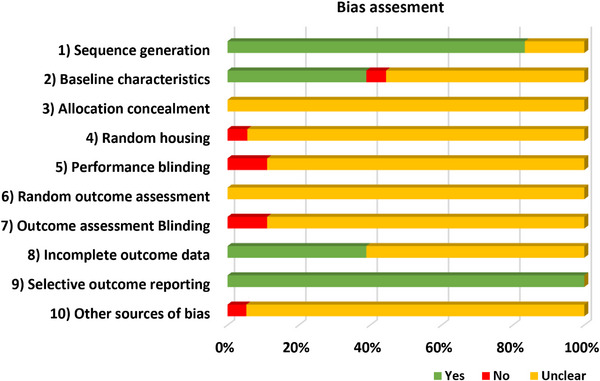
Risk of bias assessment. The 10 items detect bias related to selection (items 1–3), performance (items 4 and 5), detection (items 6 and 7), attrition (item 8), reporting (item 9) and other (item 10). Yes, demonstrates a low risk of bias; No, indicates a high risk of bias; Unclear, the risk of bias is unknown.

Figure 2 depicts the risk of bias in the studies that were included. Most had clear sequence generation, but many were unclear on allocation concealment, baseline characteristics, random housing, performance blinding, outcome assessment and other biases. However, reporting bias was consistently low. Overall, uncertainty was high across several bias domains.

### 3.3 Publication Bias

The Egger's test found no significant publication bias for most variables (egg production, egg weight, egg mass, Haugh unit, yolk index, yolk, albumen, shell weight and shell thickness), but detected bias for yolk colour (*p* < 0.05). However, applying the Trim‐and‐Fill method determined no need for additional studies, indicating insufficient evidence for missing studies that would alter the analysis.

### 3.4 Impact of Thyme on Productivity and Egg Quality Characteristics

Table 2 compares the control and thyme‐supplemented groups across various parameters. Thyme significantly increased egg weight, egg mass, yolk index, shell thickness and yolk colour (*p* < 0.05), but decreased albumen quantity. No significant changes were observed in egg production rate, Haugh unit, shell weight or yolk quantity (*p* > 0.05). Effect sizes were small for shell thickness, medium to large for yolk colour and very large for egg weight, egg mass, albumen and yolk index. Sensitivity analysis revealed high heterogeneity for most parameters (*I* ^2^ index and Cochrane *Q* test), except for shell weight, which showed moderate heterogeneity. However, no single study was identified as the primary source of this heterogeneity.

### 3.5 Analyses of Subgroups

Tables 3 and 4 present the results of the subgroup analysis based on the form of thyme and the duration of supplementation. The meta‐analysis showed that yolk colour improved in both the ≤ 8‐week and > 8‐week subgroups. However, significant increases in egg mass, egg weight, yolk and albumen were observed only in the subgroup with more than 8 weeks of supplementation (*p* < 0.05). Conversely, shell thickness and weight increased only in the ≤ 8 weeks subgroup (*p* < 0.05).

**TABLE 3:** Results of thyme supplementation on productive performance based on subgroup analyses.

Outcome	Variable	No. of trials	Effect size as (95% CI)	*p* value	*I* ^2^ (%)	*Q*−statistics (*p*)
**Egg production**	**Duration of supplementation**					
	≤ 8 weeks	7	0.29 (−1.45, 2.03)	0.743	0.0	0.493
	> 8 weeks	15	2.47 (−5.09, 10.03)	0.522	99.6	< 0.001
	**Form of thyme**					
	Essential oil	4	−1.49 (−6.31, 3.34)	0.546	78.4	0.003
	Powder	15	2.99 (−4.69, 10.67)	0.445	99.6	< 0.001
	Extract	3	1.94 (−4.15, 8.03)	0.601	0.0	0.474
**Egg weight**	**Duration of supplementation**					
	≤ 8 weeks	9	1.46 (−0.30, 3.21)	0.104	97.3	< 0.001
	> 8 weeks	17	1.25 (0.63, 1.88)	< 0.001	96.3	< 0.001
	**Form of thyme**					
	Essential oil	4	2.88 (−0.37, 6.13)	0.082	86.1	< 0.001
	Powder	19	1.15(0.16, 2.14)	0.023	99.1	< 0.001
	Extract	3	1.77 (−0.13, 3.67)	0.062	73.0	0.025
**Egg mass**	**Duration of supplementation**					
	≤ 8 weeks	9	1.79 (0.80, 2.78)	0.124	92.5	< 0.001
	> 8 weeks	11	1.26 (0.47, 2.04)	0.002	80.9	< 0.001
	**Form of thyme**					
	Essential oil	1	1.66 (−2.77, 6.10)	0.463	—	—
	Powder	17	1.79 (0.69, 2.88)	0.001	91.3	< 0.001
	Extract	2	1.72 (−0.55, 3.98)	0.138	60.6	0.111

**TABLE 4:** Results of thyme supplementation on egg quality traits based on subgroup analyses.

Outcome	Variable	No. of trials	Effect size (95% CI)	*p* value	*I* ^2^ (%)	*Q*‐statistics (*p*)
**Haugh unit**	**Duration of supplementation**					
	≤ 8 weeks	7	1.71 (−0.97, 4.39)	0.210	62.9	0.013
	> 8 weeks	15	−0.34 (−1.59, 0.90)	0.591	87.2	< 0.001
	**Form of thyme**					
	Essential oil	4	−0.80 (−4.81, 3.22)	0.697	90.9	< 0.001
	Powder	16	0.16 (−1.00, 1.31)	0.788	82.3	< 0.001
	Extract	2	0.64 (−20.04, 21.35)	0.950	96.0	< 0.001
**Yolk index**	**Duration of supplementation**					
	≤ 8 weeks	2	1.55 (−2.08, 5.18)	0.402	100.0	< 0.001
	> 8 weeks	8	2.35 (−0.46, 5.16)	0.101	98.1	< 0.001
	**Form of thyme**					
	Essential oil	1	0.10 (−0.76, 0.96)	0.820	—	—
	Powder	10	2.44 (0.75, 4.12)	0.005	99.9	< 0.001
	Extract	0	—	—	—	—
**Yolk**	**Duration of supplementation**					
	≤ 8 weeks	2	−0.98 (−3.33, 1.37)	0.413	0.00	0.525
	> 8 weeks	9	1.50 (0.12, 2.89)	0.034	95.7	< 0.001
	**Form of thyme**					
	Essential oil	1	−2.41 (−3.08, −1.75)	< 0.001	—	—
	Powder	10	1.76 (0.89, 2.62)	< 0.001	84.6	< 0.001
	Extract	0	—	—	—	—
**Yolk colour**	**Duration of supplementation**					
	≤ 8 weeks	5	0.76 (0.03, 1.49)	0.041	93.1	< 0.001
	> 8 weeks	10	0.54 (0.23, 0.85)	< 0.001	91.9	< 0.001
	**Form of thyme**					
	Essential oil	2	0.55 (−0.01, 1.10)	0.052	25.1	0.248
	Powder	11	0.80 (0.51, 1.09)	< 0.001	81.4	< 0.001
	Extract	2	0.09 (0.02, 0.16)	0.013	5.1	0.305
**Albumin**	**Duration of supplementation**					
	≤ 8 weeks	2	−0.74 (−3.07, 1.59)	0.533	0.0	0.940
	> 8 weeks	9	−1.99 (−3.02, −0.96)	< 0.001	86.6	< 0.001
	**Form of thyme**					
	Essential oil	1	−0.33 (−1.44, 0.78)	0.560	—	—
	Powder	10	−2.05 (−3.07, −1.04)	< 0.001	83.4	< 0.001
	Extract	0	—	—	—	—
**Shell thickness**	**Duration of supplementation**					
	≤ 8 weeks	5	0.04 (0.02, 0.05)	< 0.001	0.0	0.776
	> 8 weeks	15	0.01 (−0.001, 0.01)	0.073	70.8	< 0.001
	**Form of thyme**					
	Essential oil	3	0.02 (−0.01, 0.04)	0.244	87.2	< 0.001
	Powder	15	0.01 (−0.001, 0.02)	0.084	69.4	< 0.001
	Extract	2	0.04 (0.02, 0.06)	< 0.001	0.0	0.533
**Shell weight**	**Duration of supplementation**					
	≤ 8 weeks	5	−0.24 (−0.36, −0.12)	< 0.001	0.0	0.968
	> 8 weeks	7	0.01 (−0.06, 0.06)	0.908	0.0	0.950
	**Form of thyme**					
	Essential oil	2	0.07 (−0.18, 0.32)	0.565	0.0	0.754
	Powder	8	−0.03 (−0.28, 0.22)	0.823	0.0	0.971
	Extract	2	−0.11 (−0.35, 0.12)	0.339	91.7	0.001

The meta‐analysis revealed that the powdered form of thyme led to significant increases in egg weight, egg mass and yolk index, while decreasing albumen (*p* < 0.05). Both the extract and powder forms of thyme enhanced yolk colour (*p* < 0.05). The yolk quantity increased significantly with both the essential oil and powder forms of thyme (*p* < 0.05). However, shell thickness increased only with the extract form of thyme.

## 4 Discussion

This systematic review and meta‐analysis offer important insights into how dietary supplementation with *Thymus vulgaris* affects the productivity and egg quality traits of laying hens. The results indicate that thyme supplementation can significantly improve several key parameters, including egg weight, egg mass, yolk index, shell thickness and yolk colour, while reducing albumen quantity.

Thyme supplementation demonstrated a significant positive effect on egg weight and egg mass, which are critical indicators of laying hen productivity. The very large effect sizes observed for these parameters suggest that thyme can enhance the efficiency of feed conversion into egg production. This is consistent with previous studies that have highlighted the role of thyme's bioactive compounds, such as thymol and carvacrol, in improving nutrient absorption and gut health, thereby supporting better productive performance. These phenolic compounds possess potent antimicrobial activity that selectively inhibits pathogenic bacteria while promoting beneficial microbiota, thereby enhancing gut integrity and nutrient absorption efficiency. Improved gut health facilitates better digestion and assimilation of nutrients. Furthermore, the antioxidant properties of these compounds reduce oxidative stress in the intestinal mucosa and systemic circulation, supporting overall metabolic health and laying performance (Faraji et al. 2020; Gholami‐Ahangaran et al. 2022; Safavipour et al. 2022). However, no significant changes were observed in the egg production rate, which may indicate that thyme's primary benefits lie in enhancing the quality and weight of eggs rather than increasing the quantity of eggs produced.

The improvement in yolk colour, yolk index and shell thickness with thyme supplementation is particularly noteworthy. Yolk colour is a key factor influencing consumer preference, as it is often associated with the nutritional value and freshness of eggs (Chen et al. 2023; Fluck et al. 2023). The medium to large effect size for yolk colour suggests that thyme can effectively enhance this trait, likely due to its antioxidant properties and the presence of carotenoids in thyme, which can be transferred to the yolk (Kljak et al. 2021). Similarly, the increase in shell thickness is crucial for reducing egg breakage during handling and transportation, thereby improving the economic value of eggs (Mahmoud et al. 2022).

The notable decrease in albumen levels, despite no change in the Haugh unit, indicates that thyme may affect the composition of the egg white without sacrificing its freshness or quality. This finding warrants further investigation to understand the underlying mechanisms and potential implications for egg functionality in food processing.

The subgroup analyses revealed that the form and duration of thyme supplementation play a critical role in determining its efficacy. Powdered thyme was particularly effective in increasing egg weight, egg mass and yolk index, while essential oil and extract forms showed benefits for yolk colour and yolk quantity. These differences may be attributed to variations in the bioavailability and concentration of bioactive compounds in different thyme forms (Lorenzo et al. 2019; Picos‐Salas et al. 2021). The essential oils may have higher concentrations of thymol and carvacrol, which could explain their stronger impact on yolk colour (Gholami‐Ahangaran et al. 2022).

The duration of supplementation also influenced the outcomes, with longer supplementation periods (> 8 weeks) showing more pronounced effects on egg mass, egg weight and yolk quantity. This suggests that prolonged exposure to thyme's bioactive compounds is necessary to achieve significant improvements in these parameters. Conversely, shell thickness and weight were more responsive to shorter supplementation periods (≤ 8 weeks), indicating that these traits may be influenced by early‐phase physiological changes in the hens.

The findings of this study have important implications for the poultry industry, particularly in the context of increasing consumer demand for natural and sustainably produced eggs. Thyme supplementation offers a viable strategy for enhancing egg quality traits such as yolk colour and shell thickness, which are critical for consumer appeal and marketability. Moreover, the use of thyme as a phytogenic additive aligns with the industry's shift away from synthetic additives, addressing concerns related to antibiotic resistance and food safety (Gadde et al. 2017; Lillehoj et al. 2018). However, the choice of thyme form and supplementation duration should be carefully considered to maximize its benefits. Powdered thyme may be more suitable for improving egg weight and mass, while essential oils or extracts could be preferred for enhancing yolk colour. In addition, producers should consider the cost‐effectiveness and practicality of different thyme forms in large‐scale operations.

While this study provides comprehensive insights into the effects of thyme supplementation, many limitations should be acknowledged. Firstly, the high heterogeneity observed for some parameters, such as egg production rate and yolk colour, suggests variability in study designs, dosages and experimental conditions. Future research should aim to standardize these factors to reduce heterogeneity and improve the reliability of meta‐analytic findings. Secondly, the risk of bias assessment revealed uncertainties in several domains, particularly in performance and detection bias. This highlights the need for more rigorous experimental designs and transparent reporting in future studies. In addition, there is potential for publication bias, particularly for yolk colour.

## 5 Conclusions

This systematic review and meta‐analysis show that adding *Thymus vulgaris* to the diet of laying hens significantly improves egg quality, such as egg weight, egg mass, yolk colour, yolk index and shell thickness. The form and duration of thyme supplementation are important for its effectiveness, with powdered thyme and longer use showing the best results. These findings support using thyme as a natural alternative to synthetic additives in poultry feed, which aligns with consumer demand for environmentally friendly products.

## Author Contributions

**Hossein Hassanpour**: conceptualization, investigation, writing – original draft, methodology, validation, visualization, software, formal analysis, data curation, supervision, writing – review and editing. **Leila Nasiri**: methodology, writing – original draft, formal analysis, data curation, writing – review and editing. **Aziz A. Fallah**: validation, visualization, software. **Tahereh Karimi‐Shayan**: validation, data curation, formal analysis.

## Ethics Statement

The authors have nothing to report.

## Conflicts of Interest

The authors declare no conflicts of interest.

## Peer Review

The peer review history for this article is available at https://www.webofscience.com/api/gateway/wos/peer‐review/10.1002/vms3.70614.

## Data Availability

Data are available upon request.
